# HIV-Exposed Uninfected Infants Have Increased Regulatory T Cells That Correlate With Decreased T Cell Function

**DOI:** 10.3389/fimmu.2019.00595

**Published:** 2019-03-26

**Authors:** Emilie Jalbert, Kayla M. Williamson, Miranda E. Kroehl, Michael J. Johnson, Clare Cutland, Shabir A. Madhi, Marta C. Nunes, Adriana Weinberg

**Affiliations:** ^1^University of Colorado Denver Anschutz Medical Center, Aurora, CO, United States; ^2^University of Witwatersrand, Johannesburg, South Africa

**Keywords:** HIV-exposed uninfected infants, regulatory T cells, T cell function, antigen presenting cells, T cell differentiation

## Abstract

**Background:** HIV-exposed uninfected infants (HEU) are at higher risk of severe infections, hospitalizations and death compared with HIV-unexposed uninfected infants (HUU), but the immune deficit underlying it is not known. To address this gap, we investigated T cell functionality and its relationship to phenotypic profiles of T cells and antigen presenting cells (APC) in HEU and HUU.

**Methods:** Blood mononuclear cells from 55 HEU and 16 HUU were stimulated with Staphylococcal Enterotoxin B (SEB) or mock for 72 h, and tested by flow cytometry for proliferation and expression of Th1, Th2, and regulatory (Treg) markers. In parallel, cells were phenotypically assessed for differentiation profiles of Treg, conventional T cell (Tconv) and APC in unstimulated cells.

**Results:** HEU had lower CD4+ functional responses to SEB/mock and similar CD8+ responses compared with HUU. In the phenotypic T cell panel, HEU showed higher proportions of CD4+ and CD8+ Treg expressing IL10, FOXP3, and CD25; higher effector Tconv and Treg; and lower naïve and CD4+TGFβ+ Treg compared with HUU. In the phenotypic APC panel, HEU showed higher proportions of CD1c+ cDC2, CD123+ pDC, CD16+ inflammatory monocytes and cDC and higher expression of CD103 on CD1c-CD123-CD16-cDC1 compared with HUU. Regression analyses adjusted for HIV exposure and multiple comparisons showed that higher CD8+IL10+ and CD8+FOXP3+ Treg in unstimulated cells were associated with lower CD8+ T cell functional responses to SEB/mock. Functionality was not affected by Tconv differentiation, but higher APC activation in aggregate was associated with higher CD8+IL10+ Treg responses to SEB.

**Conclusions:** T cell functionality was decreased in HEU compared with HUU. High CD8+ Treg proportions were the most important predictors of decreased T cell functionality in HEU and HUU.

## Introduction

Infants are at higher risk of severe infections and have lower immune responses to most vaccines than older children and young adults. This has been ascribed to an imbalance of T-helper (Th) Th1 and Th2 immune responses in infants (1–3). Neonates, however, also have strong immune regulatory mechanisms that promote their survival *in utero* while surrounded by foreign maternal antigens (4–7). An example of the role of neonatal regulatory T cells (Treg) in the risk of infectious morbidity is provided by the use of cord blood in allogeneic hematopoietic stem cell transplantation, which, compared with adult cell transplants, has been associated with higher risk of opportunistic infections (8, 9). Moreover, adaptive T-cell responses to foreign antigens that cross the placenta can be elicited using Treg-depleted cord blood mononuclear cells (CBMC) but not with undepleted CBMC (10). Collectively, these data indicate that it is reasonable to propose that high proportions of Treg may be associated with increased severity of infections in infants.

HIV-exposed uninfected infants (HEU) have a significantly higher incidence of severe infections, hospitalizations and death (11–24) and lower immune responses to some vaccines (25–31) than HIV-unexposed uninfected infants (HUU). Much of the excess morbidity and mortality of HEU is due to severe infections caused by respiratory viral pathogens and *S. pneumoniae* (24, 28, 31–33). It has been demonstrated that HEU generally have lower maternal antibodies against many of these pathogens compared with HUU (26, 31, 34). However, we recently found that antibody titers against respiratory viruses or *S. pneumoniae* in the first few days of life were not associated with the development of lower respiratory tract infections in HEU (35). Furthermore, antibody responses to tetanus vaccine also failed to discriminate between HEU who developed lower respiratory tract infections or not underscoring the lack of association between humoral immune responses and risk of severe infections in HEU (35). Collectively, these data suggested that defective T cell or innate immune responses may be primarily responsible for the morbidity and mortality of infections in HEU. The pathway leading to cellular immune defects in HEU is not known (25, 27, 36–46), but excessive immune regulation is a potential unifying explanation for the diverse immune defects of HEU, since Treg and other regulatory cells decrease both innate and adaptive immune responses (47–49). Pregnant women and other people living with HIV have higher markers of activation, inflammation and regulation than their uninfected counterparts. HEU also have higher levels of inflammation and T cell and dendritic cell (DC) activation compared with HUU (36, 50, 51). However, until now, there have been no published studies comparing Treg between HEU and HUU. Moreover, the effect of T cell and DC activation on functional T cell responses has not been studied.

To address this gap and to expand our understanding of the immunologic differences between HEU and HUU we performed hypotheses-generating analyses of T cells and antigen presenting cells (APC) in peripheral blood mononuclear cells (PBMC) collected in the first 1–2 days of life from HEU and in CBMC of HUU. In order to generate mechanistic hypotheses, we placed special emphasis on the relationship between functional T cell responses and phenotypic T cell and antigen presenting cell (APC) characteristics.

## Samples and Methods

### Samples

The study used a convenience sample of PBMC collected in the first 48 h of life in a previous study from 55 Black South African HEU, including 42% females and 86% term infants. The legal guardians of study participants signed informed consents and the Ethics Committee of the Witwatersrand University approved the use of the study. In lieu of HUU PBMC, we used CBMC from 16 HUU about to be discarded by the cord blood bank at the University of Colorado Denver Anschutz Medical Campus. The CMBC were obtained from term infants, including 50% White Caucasians, 44% non-Black Hispanics and 56% females. Due to limited number of PBMC from HEU, not all assays could be performed on each participant. In order to obtain roughly similar number of results for each test, we used a priority list among T cell functional, T cell phenotypic, and APC phenotypic assays that changed with every batch of HEU samples tested. PBMC included in each batch were selected in the order of the study identification numbers.

### Flow Cytometry Methods

Cryopreserved CBMC/PBMC were thawed and processed immediately for phenotypic and functional assessment. Phenotypic profiling was performed using flow cytometry by staining with either the APC or the T cell panel. The APC panel consisted of surface staining with Zombie yellow (viability), CD14 Alexa488, CD103 PE, CD123 PerCP-Cy5.5, CD1c APC, PD-L1 BV421 (Biolegend) and CD16 PE-CF594, CD3 PE-Cy7, CD56 PE-Cy7, CD19 PE-Cy7, CD20 PE-Cy7, CD40 Alexa700, HLA-DR APC-H7 (BD Biosciences). The T cell panel consisted of surface staining with Zombie yellow (viability), CD28 PE-Dazzle594, CD27 BV421 (Biolegend) and CD39 FITC, CD4 PerCP-Cy5.5, CD25 APC-H7, CD3 Alexa700 (BD Biosciences) followed by fixation and permeabilization using the eBioscience Foxp3/Transcription Factor Staining Buffer Set. Intracellular staining was then performed with FoxP3 PE (eBioscience) and IL-10 PE-Cy7, TGFβ APC (Biolegend).

For the functional assay, cells were first stained with cell trace violet (LifeTechnologies) then stimulated for 72 h with Staphylococcal Enterotoxin B (SEB) or with no stimulation (media only). Brefeldin A and monensin (Sigma-Aldrich, 5 μg/ml each) were added to the cultures for the last 4 h. Cells were then surface stained with Zombie yellow (viability), CD39 FITC and CD4 PerCP-Cy5.5 (BD Biosciences) followed by fixation (BD lysing solution) and permeabilization (BD perm2 buffer) and stained intracellularly with IL-4 PE, CD3 Alexa700, IFNγ APC-H7 (BD Biosciences), and IL-10 PE-Cy7, TGFβ APC (Biolegend).

Samples were acquired on a Beckman Coulter Gallios cytometer and analyzed with FlowJo version 9.0 (BD Biosciences). Gating strategies are shown in Figure S1.

### Statistical Methods

Frequencies (%) or means and standard deviations were calculated for baseline demographics. Heatmaps were drawn to show the Spearman correlation between cell populations. Correlations between HEU and HUU were assessed using simple linear regression models where cell populations were primary outcomes and maternal HIV status was the main covariate. Multiple linear regression models were used to assess relationships between various cell populations adjusting for maternal HIV status. Cell population distributions were examined using graphical inspection and log transformations were used when distributions were highly skewed. To account for multiple comparisons, a false discovery rate (FDR) correction was implemented for each set of comparisons among cell populations, and significance was evaluated at a FDR p-threshold of 0.05. Principal component analysis (PCA) using Euclidean distances were drawn for functional differences in SEB and no stimulation for CD4 and CD8 to evaluate potential differences in populations between HUU and HEU. Further, the first 3 PCs for the activation markers and CD4/CD8 T cell subsets were tested via linear regression for association with functional differences for CD4 and CD8. Functional outcomes were log transformed and the regression coefficients were back transformed to the original scale. Data analysis was performed using R software, version 3.4.0.

## Results

### Functional T Cell Characteristics of HEU and HUU

**Figure 1:**
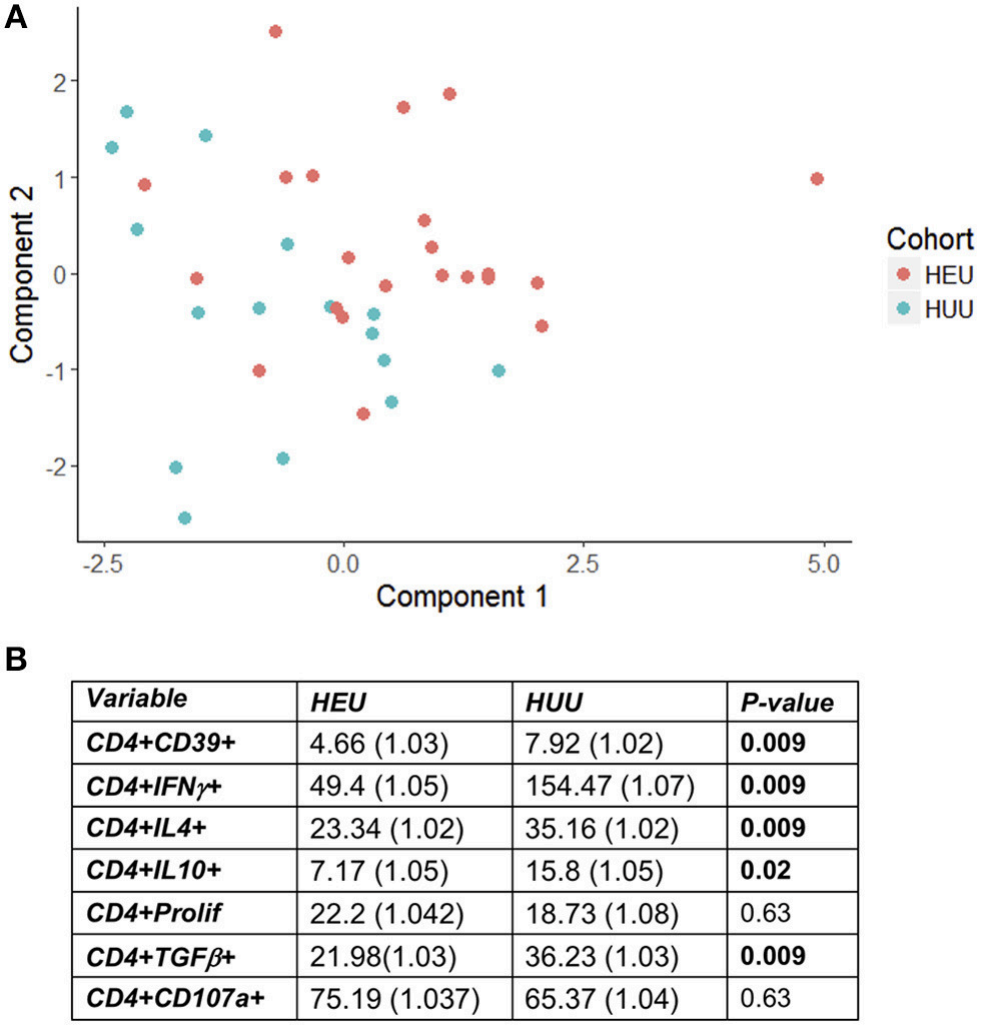
HEU and HUU functional CD4+ T cell responses. Data were derived from PBMC of 22 HEU and 17 HUU after stimulation with SEB and mock. Results were expressed as the ratio between SEB and mock stimulation. **(A)** shows the PCA distribution of the results. **(B)** shows results of the regression analysis.

**Figure 2:**
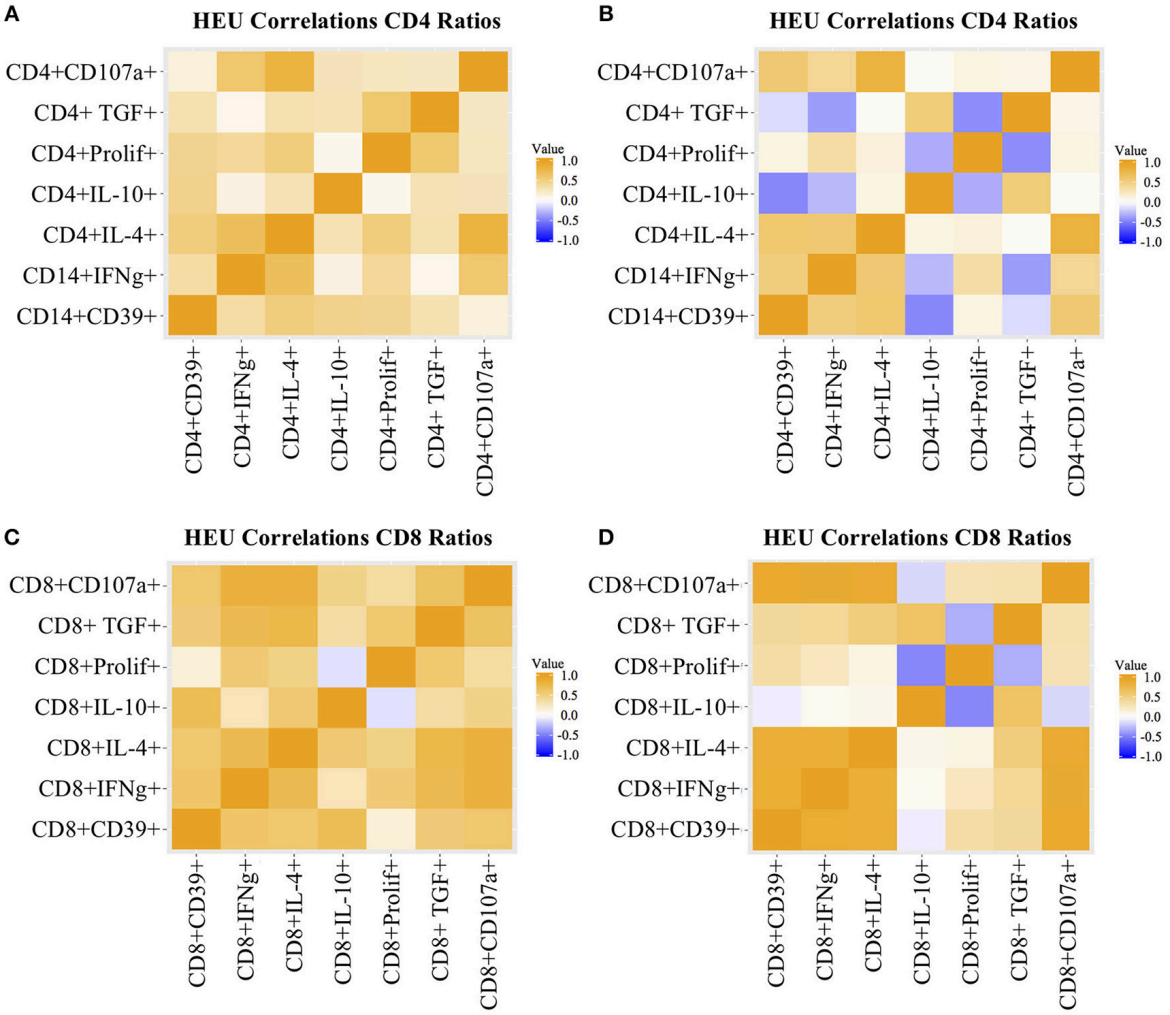
Heatmap representation of functional T cell response correlations. Data were derived from PBMC of 22 HEU and 17 HUU after stimulation with SEB and mock. Analytes are shown on the graph axes. The correlation factor scale is shown on the side of each heatmap. The data represented in panels **(A–D)** are labeled in each of the corresponding panels.

T cell proliferation and expression of Th1 (IFNγ, CD107a), Th2 (IL4), and Treg (IL10, TGFβ, and CD39) were investigated by stimulating PBMC from 22 HEU and 17 HUU with SEB and mock control and expressed as the ratio of stimulated over mock PBMC. The PCA visualization of the data (Figure 1A) showed separation of the CD4+ T cell outcome measures between HEU and HUU, but not of CD8+ T cell outcomes (Figure S2A). The linear regression analysis revealed significantly lower expression of IFNγ, IL4, IL10, TGFβ, and CD39 in HEU compared to HUU CD4+ T cells in response to SEB stimulation (Figure 1B), but not in CD8+ T cells (Figure S2B). Correlation analyses revealed strong positive associations (rho > 0.8) between CD4+CD107a+ and CD4+IL4+ responses to SEB in HEU and HUU (rho = 0.81 for both; Figures 2A,B). Similar analyses of CD8+ T cell responses to SEB showed strong positive correlations of CD8+CD107a+ with CD8+IL4+ and CD8+IFNγ+ in HEU (rho ≥ 0.81; Figure 2C) and strong positive correlations of CD8+ T cell expression of CD39, CD107, IFNγ, IL4 and/or IL10 in HUU (rho ≥ 0.81; Figure 2D). Importantly, there were no significant negative correlations between Treg responses to SEB and Th1 or Th2 responses to SEB.

### T Cell Phenotypic Characterization

We performed multiple pre-specified comparisons of the T cell phenotypes of HEU and HUU, including 28 Treg and Tconv subsets. Overall, the ratios of CD4+ to CD8+ T cell populations were similar in HEU and HUU. However, the proportions of multiple CD4+ and CD8+ T cell subsets significantly differed in unadjusted comparisons (Figure S3), 10 of which remained significantly different after FDR correction (Table 1). These included CD4+/CD8+FOXP3+, CD4+/CD8+FOXP3+CD25+, and CD8+IL10+ Treg, which were higher in HEU compared with HUU; CD4+TGFβ+ Treg, lower in HEU; CD8+FOXP3+CD27+CD28- and CD8+FOXP3+CD27-CD28-differentiated Treg, and CD8+CD27+CD28-differentiated Tconv higher in HEU; and CD8+FOXP3+CD27+CD28+ naïve Treg higher in HUU.

**Table 1:** T cell phenotypic subsets significantly different between 26 HEU and 17 HUU.

**Subset**	**HEU**	**HUU**	***P*-value**
CD4+FoxP3+	5.18 (0.08)^*^	2.34 (0.09)	0.0004^#^
CD4+TGFβ+	0.15 (0.003)	0.30 (0.01)	0.01
CD8+FoxP3+	1.27 (0.05)	0.20 (0.009)	0.01
CD8+FoxP3+CD25+	0.04 (0.0008)	0.02 (0.001)	0.01
CD8+L10+	0.31 (0.005)	0.13 (0.006)	0.0001
CD8+FoxP3+CD27+CD28+	48.1 (0.8)	77.7 (0.78)	0.0001
CD8+FoxP3+CD27+CD28-	29.7 (0.57)	16.5 (0.56)	0.01
CD8+FoxP3+CD27-CD28-	21.3 (0.56)	5.02 (0.47)	0.002
CD8+CD27+CD28-	8.51 (0.34)	3.02 (0.13)	0.046

* *Numbers represent mean (SEM)*.

# *P-values were calculated by linear regression and adjusted for multiple comparisons by FDR*.

### Phenotypic Characterization of APC

The unadjusted analyses identified multiple APC phenotypic differences in HEU compared with HUU (Figure S4). Overall, HEU had higher proportions of CD16+ Mono, CD16+CD123-CD1c- DC (CD16+ cDC) and CD123+ pDC compared with the HUU, but lower proportions of CD16- Mono (Mono1) and CD123-CD1c-CD16- DC (cDC1). HEU also had increased PDL1 expression on cDC1, cDC2, pDC, and Mono1 and increased expression of CD103 on cDC1 and cDC2. After FDR correction, the following observations remained significantly different: higher proportions of pDC, CD16+ cDC and cDC2 and CD16+ Mono out of APC, and higher CD103 expression on cDC1 in HEU compared with HUU (Table 2).

**Table 2:** APC subsets significantly different between 27 HEU and 17 HUU.

**Subset**	**HEU**	**HUU**	***P*-value**
16+ cDC	1.37 (1.05)^*^	0.15 (1.07)	<0.001^#^
cDC2	1.60 (1.03)	0.82 (1.04)	0.006
pDC	2.83 (1.03)	1.07 (1.03)	<0.001
CD16+ Mono	6.69 (1.03)	3.32 (1.05)	0.02
cDC1 CD103+	2.03 (0.04)	0.60 (0.02)	0.0001

* *Numbers represent mean (SEM)*.

# *P-values were calculated by linear regression and adjusted for multiple comparisons by FDR*.

### Correlation of T Cell Function With Treg and T Cell Differentiation Phenotypes

**Figure 3:**
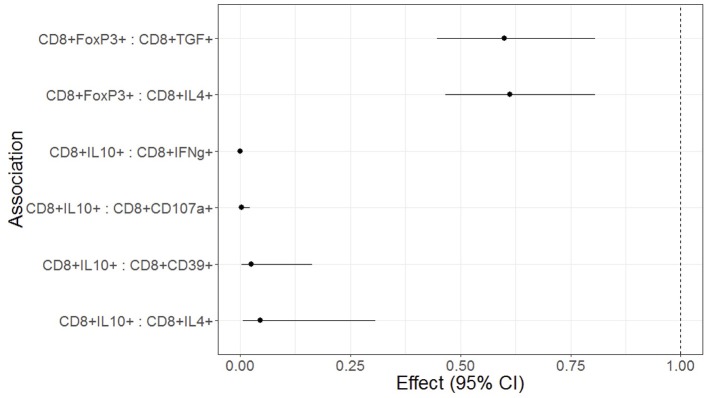
Forest plot representation of significant effects of Treg on functional T cell outcomes in HEU and HUU. Data were derived from 6 HEU and 16HUU. Filled circles and horizontal lines indicate mean and 95% CI of the effect of each Treg population on the functional outcome indicated on the abscissa (Treg : functional outcome). No effect is indicated by the dotted vertical line. The effect represents the decrease of the functional outcome with each unit increase in the Treg population. For example, for each 1% increase in CD8+FOXP3 Treg, there is a 40% decrease in the CD8+TGFβ T cells; and for each 1% increase in CD8+IL10+ Treg there is a 95% decrease in the CD8+IFNγ+ T cells.

We investigated the effect of Treg, Tconv, and APC phenotypes on functional T cell responses to SEB stimulation in a regression analysis adjusted for maternal HIV status and for multiple comparisons. High frequencies of circulating CD8+IL10+ Treg were significantly associated with low CD8+IFNγ+, CD8+CD107a+, CD8+CD39+, and CD8+IL4+ responses to SEB stimulation (Figure 3). High frequencies of circulating CD8+FOXP3+ Treg were associated with low CD8+IL4+ and CD8+TGFβ+ responses to SEB stimulation (Figure 3). CD4+ Treg, CD4+ or CD8+ T cell differentiation and APC phenotypes were not associated with T cell responses to SEB (not shown).

### Correlation of T Cell Function With APC Activation

None of the individual activation markers expressed by DC or Mono subsets showed significant effects on T cell functional outcomes. However, we reasoned that the high number of explanatory variables might have obscured significant associations. To decrease the dimensionality of the activated APC subset, we grouped them using PCA (Table 3). 95.7% of the variation of the activated APC subsets was explained by the first 3 PCs with 63% of the variation explained by PC1. The correlation analysis of PC1 with T cell functional outcomes revealed a significant positive association with CD8+IL10+ responses to SEB (effect estimate ±S.E. = 1.02 ± 1.01; *p* = 0.007). This indicates that PDL1+ cDC1, cDC2, pDC and CD16- Mono, as well as CD40+ cDC1 and cDC2, all moderately to highly correlated with PC1, may be codependently correlated with CD8+IL10+ responses to SEB. PC2 and PC3 did not significantly correlate with any of the functional outcome measures.

**Table 3:** Loadings from principal component analysis of activated APC.

	**PC1**	**PC2**	**PC3**
Explained Variation %	63.39	19.51	12.82
cDC2 PDL1+	0.92	0.34	−0.21
cDC1 CD40+	0.89	0.45	0.06
cDC1 PDL1+	0.79	−0.57	0.24
cDC2 CD40+	0.79	0.57	0.25
pDC PDL1+	0.73	−0.01	0.68
Mono1 PDL1+	0.52	−0.75	0.41
16-cDC CD103+	0.37	−0.61	0.70
16-cDC PDL1+	0.33	−0.94	−0.06
cDC1 CD103+	0.30	0.24	0.92
CD16-Mono PDL1+	0.28	−0.93	0.23
Mono1 CD40+	0.21	0.47	0.86
cDC2 CD103+	0.20	0.03	0.98
pDC CD103+	0.16	−0.34	0.93
Mono1 CD103+	0.11	0.18	0.98
pDC CD40+	0.06	0.64	−0.76
CD16-Mono CD103+	0.03	0.64	0.77
16-cDC CD40+	−0.66	−0.64	−0.40
CD16-Mono CD40+	−0.75	0.30	0.59

## Discussion

The primary objective of this study was to identify factors that may explain decreased CMI responses to vaccines in HEU and that might be amenable to therapeutic interventions. Our primary focus was on Treg, because of their broad immune-attenuating and sometimes nonspecific activity and because of the association of HIV infection with increased Treg populations (52–58). In addition, preliminary results obtained from 7 South African HUU and 4 HEU showed significantly higher CD4+ and CD8+ FOXP3+ and FOXP3+CD25+ Treg in HEU compared with HUU. In the current study, we demonstrated an increased proportion of both CD4+ and CD8+ Treg in HEU compared with HUU using US CBMC to generate the HUU. Although the differences between US CBMC and South African HEU PBMC were similar to those between South African HUU and HEU, the use of CBMC was a limitation of this study. For example, the difference in Treg between HEU and HUU might have been even more significant if we had used HEU and HUU cells of the same age instead of PBMC from 1 to 2 days of age from HEU and CBMC from HUU. Recent findings showed that the proportions of Treg significantly decrease between birth and the first few days of life (59), suggesting that we might have underestimated the Treg differences HEU and HUU by virtue of using samples from slightly older HEU compared with HUU.

In this study, we showed for the first time to our knowledge that HEU have significant defects in T cell functionality at birth by comparison with HUU. This observation is highly relevant to the current landscape of HIV infection, in which there is a continuously growing number of HEU with increased risk of severe infections leading to hospitalization and/or death (11–24). It is important to note that this observation, originally made in countries with limited medical resources, has been extended to countries with high resources, including the US (60). HIV infection during pregnancy has been long known to decrease transplacental transfer of maternal antibodies (31, 61, 62), potentially increasing the vulnerability of HEU to infections. However, our most recent studies showed that maternal antibody levels transferred to the neonate did not predict the risk of lower respiratory tract infections in HEU (35). Furthermore, the antibody responses to vaccines of HEU has been comparable to that of HUU in studies originated in Sub-Saharan Africa, suggesting that HEU have intact humoral immunity (34, 63). A minority of studies, all of them including participants from Brazil, reported lower antibody responses in HEU compared with HUU (28, 31). Nevertheless, in our study comparing Brazilian HEU and HUU, despite showing decreased antibody responses to tetanus vaccine in HEU, the magnitude of the HEU antibody responses to the vaccine did not correlate with the risk of developing lower respiratory tract infections during the first 6 months of life, underscoring the lack of association between humoral immunity and risk of severe respiratory tract infection in HEU (35). Taken together, these data suggest that T cell immune defects play a more important role than antibodies in the increased susceptibility to infection of HEU.

Other studies that showed decreased T cell responses to BCG or tetanus vaccines during the first few months of life in HEU (11–24) support and complement our findings by indicating the persistence of T cell functional defects in HEU during the first year of life and possibly longer. It is also important to note that the increased risk of hospitalization and death due to infectious complications in HEU has been demonstrated for the first 1–2 years of life, further suggesting an association between decreased T cell functional responses and clinical outcomes.

The effect of increased proportions of Treg on functional responses in HEU was investigated by regression analysis adjusted for maternal HIV status. The adjustment was performed with the intent of increasing the probability of identifying causal associations. We reasoned that since HEU had both lower functional responses and higher Treg proportions, there was a considerable risk of finding both outcome measures in the same individual by chance, which we tried to prevent by controlling for the maternal HIV status. Nevertheless, the adjusted analysis identified CD8+FOXP3+ and CD8+IL10+ Treg as predictors of decreased CD8+ T cell functionality in HEU and HUU. It is important to note that the T cell functional responses that appear to be downregulated by Treg were not the same T cell functional responses that differed between HEU and HUU. This, however, was not unexpected due to the adjustment of the statistical analysis for the HIV maternal status.

We also found higher proportions of Treg and Tconv differentiated to the effector stage in HEU compared with HUU. Previous studies also showed higher level of T cell activation in HEU compared with HUU, which is in accordance with our findings, since effector T cells generally also express activation markers (36). It is well-known and accepted that T cell differentiation into effector and memory cells occurs in the presence of cognate antigen and is mediated by activated APC and lineage-specific cytokines. It is unclear what antigens triggered the T cell differentiation in HEU. However, we have previously shown higher pro-inflammatory cytokine milieu in HEU compared with HUU, including sTNFRI, IL6, and IP10 that could potentially lower the threshold of T cell activation and differentiation in HEU (50).

We also showed in this study increased expression of CD40, CD103, and PDL-1 in HEU compared with HUU in unadjusted analyses and CD103 on cDC1 after adjustment for multiple comparisons, indicating that increased activation is not limited to T cells, but also encompasses APC. None of the activated APC populations individually correlated with T cell function, but the PCA showed that activation in general, represented by either CD40 or PDL1 expression, was associated with increased CD8+IL10+ Treg proliferation in response SEB. Other functional T cell outcomes did not significantly correlate with APC.

Our study has several limitations, including the geographic and racial differences between HEU and HUU, the limited cell numbers in HEU samples, its exploratory nature and the lack of mechanistic experiments. As mentioned above, the age difference between HEU and HUU may have underestimated the Treg differences identified in our study. A strength of this study is the use of a superantigen as stimulant, which enlists the collaboration between T cells and APC into the response.

Several hypotheses can be formulated based on the results of this exploratory study. Firstly, we propose that increased Treg populations in HEU decrease their T cell responses to infections and vaccines. This hypothesis is being tested in studies that we are currently conducting. Secondly, we propose that the high inflammatory *in utero* milieu of HEU may lower the threshold for T cell activation and differentiation against antigens to which they are commonly exposed *in utero* and that do not trigger responses in HUU. Thirdly, there may be increased transplacental transfer of antigens in pregnancies complicated by HIV infection.

## Data Availability

All datasets generated for this study are included in the manuscript and/or the Supplementary Files.

## Author Contributions

EJ performed assays, analysis and wrote manuscript. KW and MK performed statistical analysis and wrote manuscript. MJ performed analysis and reviewed manuscript. CC, SM and MN enrolled study participants and reviewed the manuscript. AW designed the study, analyzed the data, and wrote the manuscript.

### Conflict of Interest Statement

The authors declare that the research was conducted in the absence of any commercial or financial relationships that could be construed as a potential conflict of interest.
