# Supplementary material for: HIV-Exposed Uninfected Infants Have Increased Regulatory T Cells That Correlate With Decreased T Cell Function
Source: Front Immunol. 2019 Mar 26;10:595. doi: 10.3389/fimmu.2019.00595 (PMC6445326; doi:10.3389/fimmu.2019.00595)
Supplement: Supplementary file 1 [file Image_1.pdf]

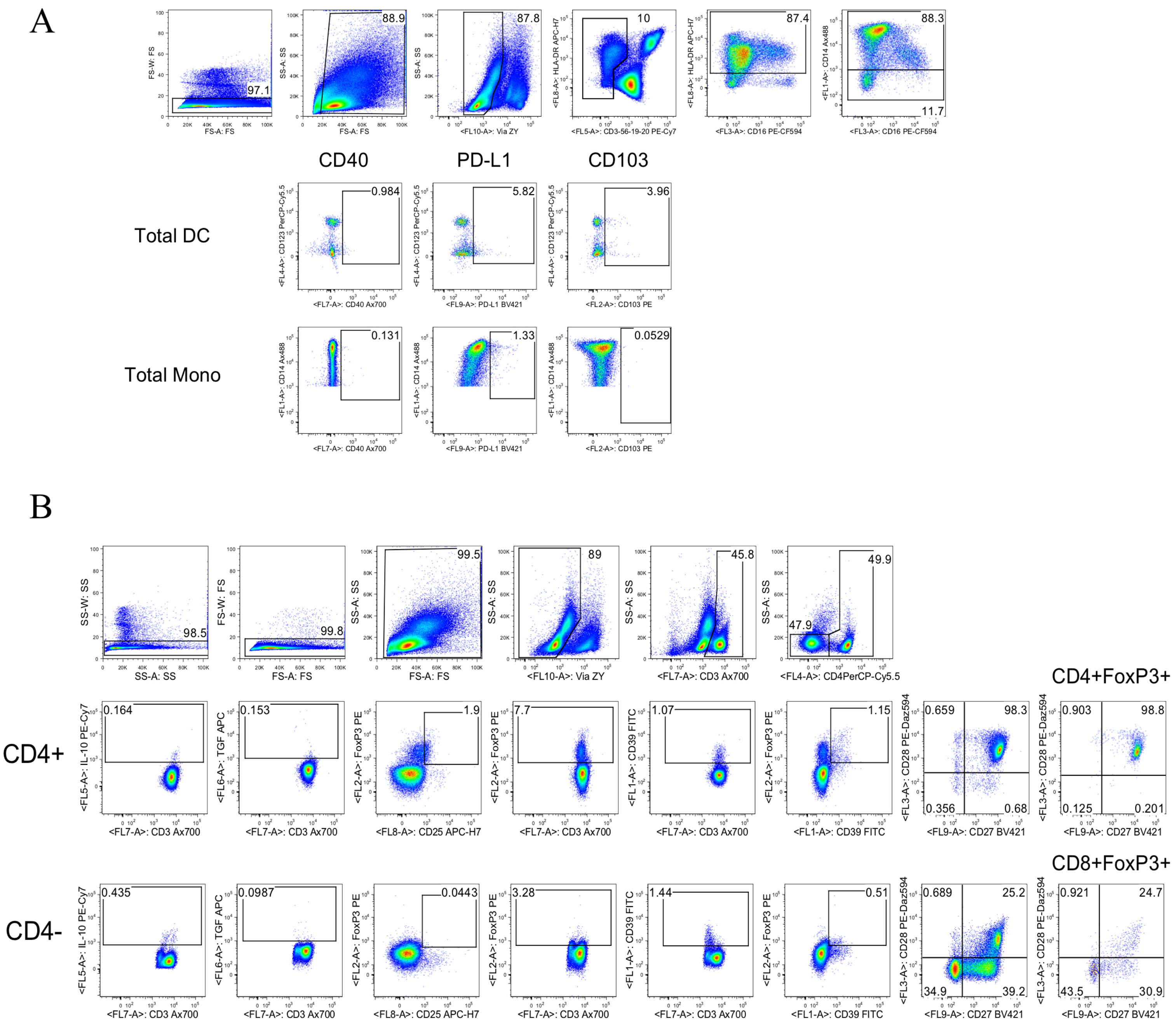

Figure S1. Gating strategies. Data were derived from one of the HEU. **Panel A** shows the phenotypic APC strategy; **Panel B** shows the phenotypic T cell strategy; and **Panel C** the functional T cell strategy.

C

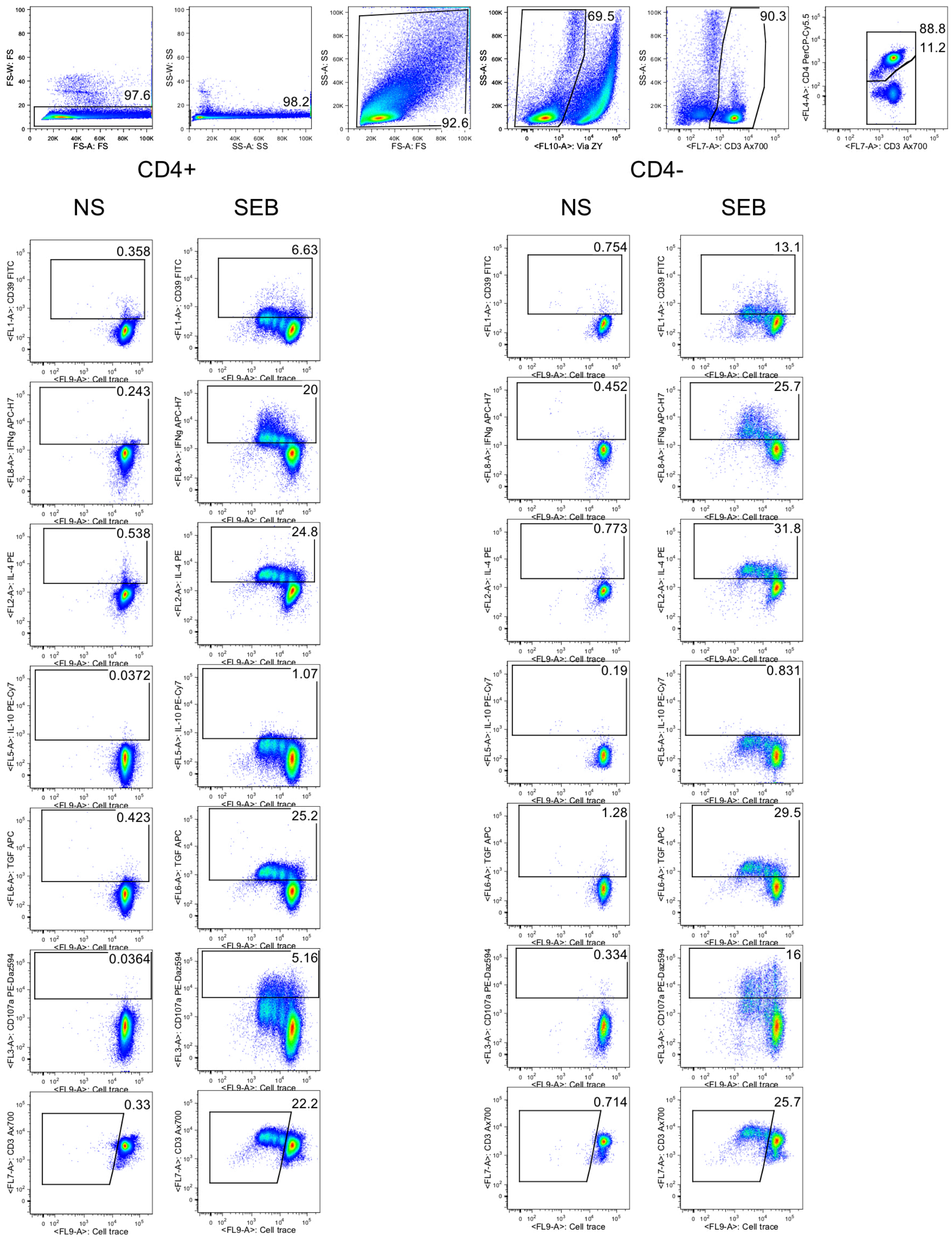

Figure S1. Gating strategies. Data were derived from one of the HEU. **Panel A** shows the phenotypic APC strategy; **Panel B** shows the phenotypic T cell strategy; and **Panel C** the functional T cell strategy.
