# Supplementary figures and images for: HIV-Exposed Uninfected Infants Have Increased Regulatory T Cells That Correlate With Decreased T Cell Function
Source: Front Immunol. 2019 Mar 26;10:595. doi: 10.3389/fimmu.2019.00595 (PMC6445326; doi:10.3389/fimmu.2019.00595)

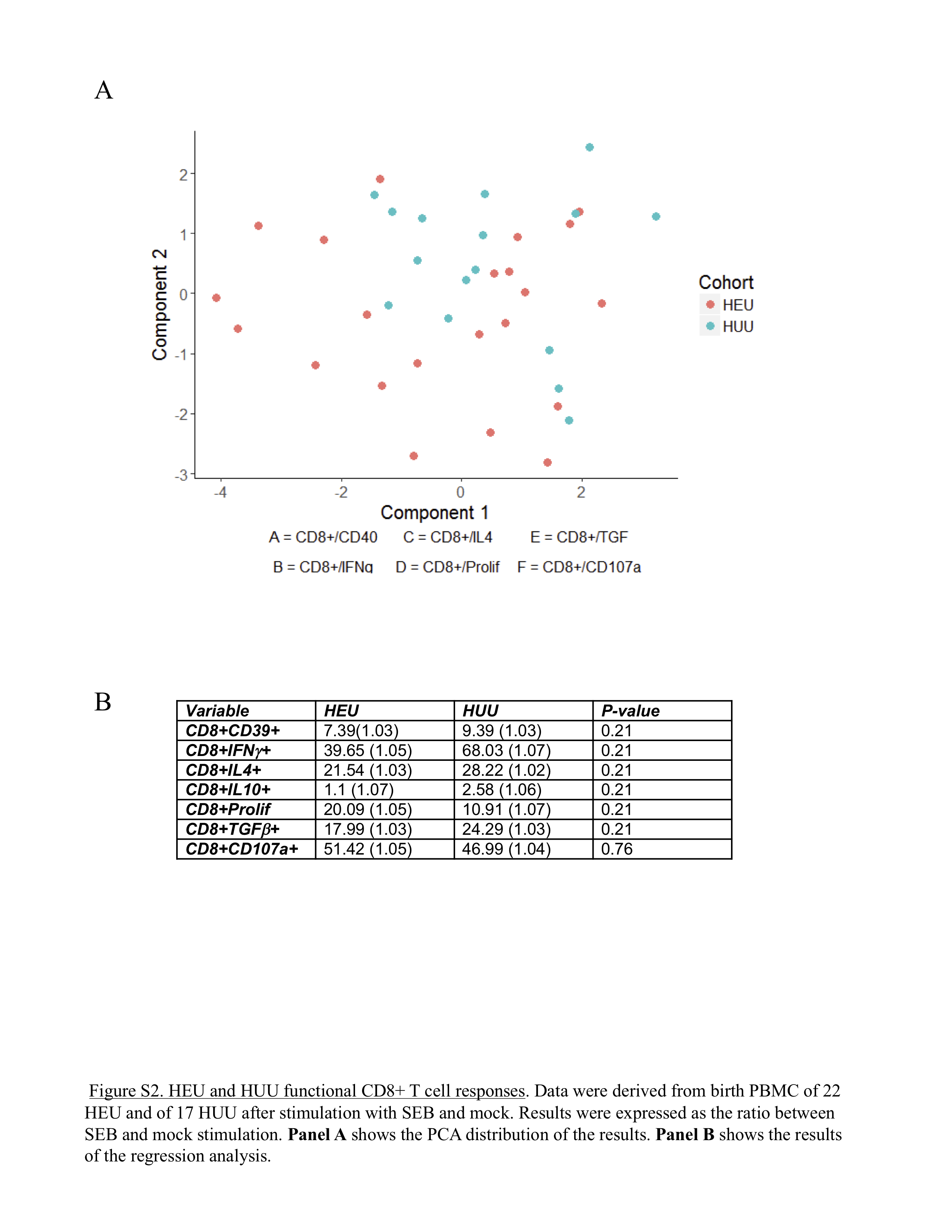

Supplement: Supplementary file 2 [file Image_2.jpeg]

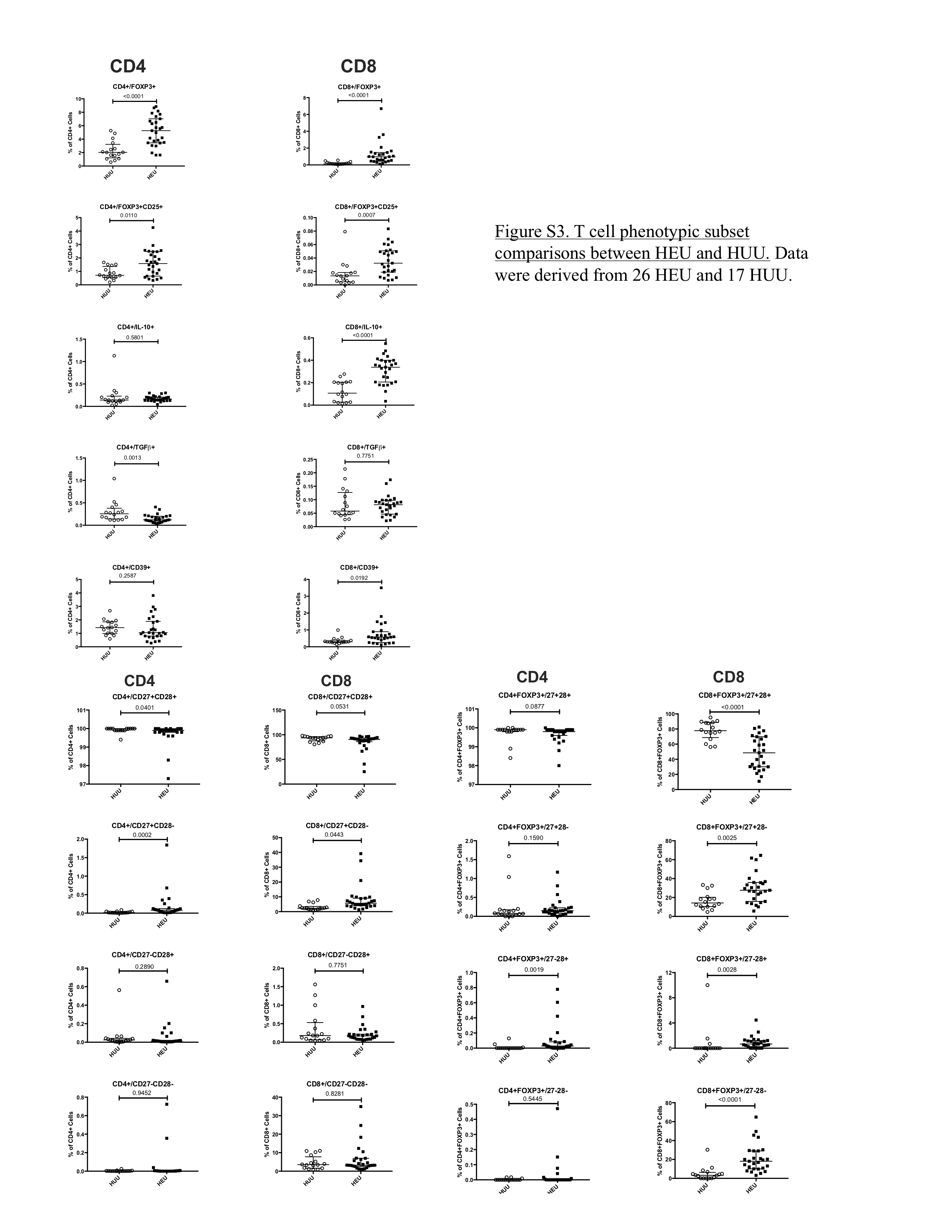

Supplement: Supplementary file 3 [file Image_3.jpeg]

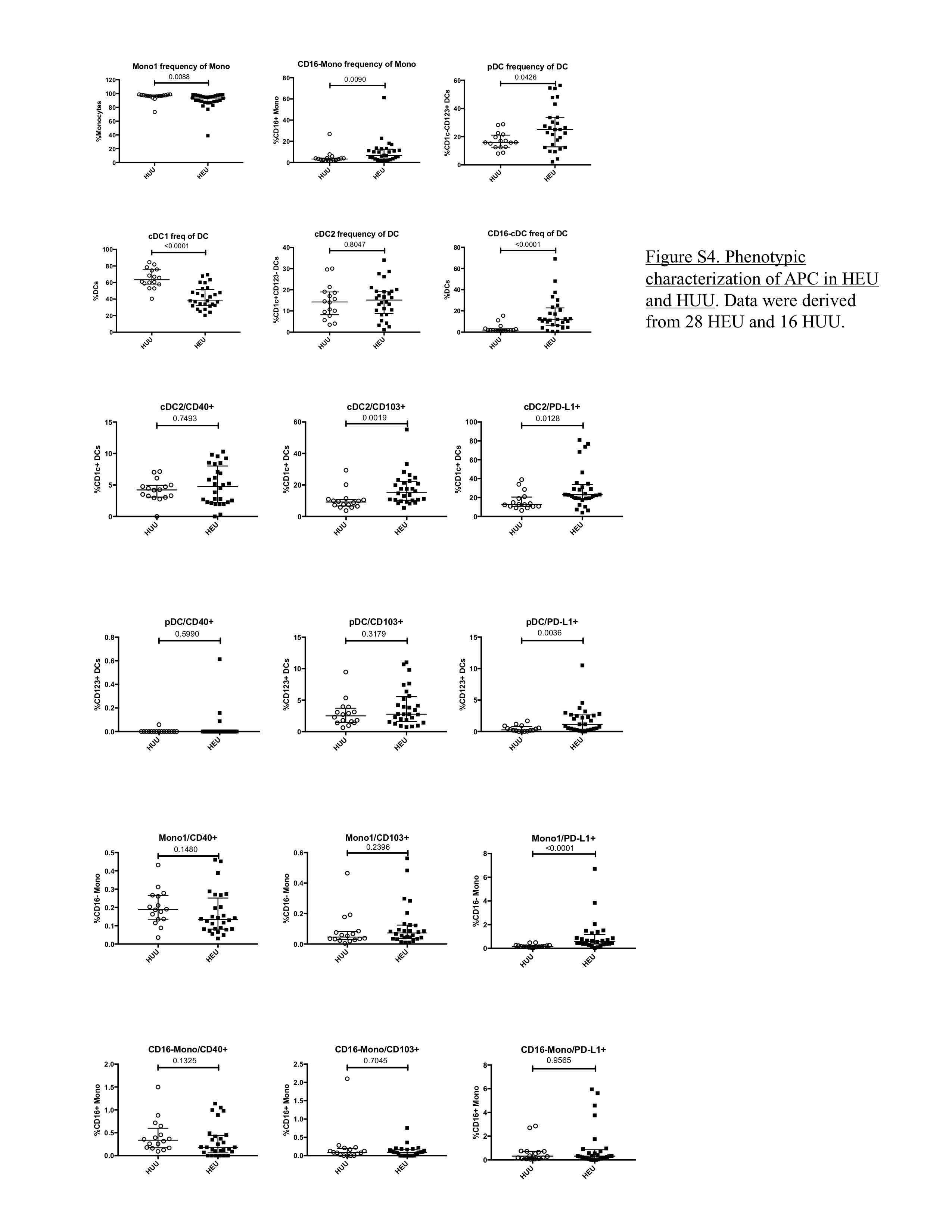

Supplement: Supplementary file 4 [file Image_4.jpeg]
